# Case report: A case of complete clinical response in a patient experiencing high microsatellite instability unresectable colon cancer being treated with a PD-L1 inhibitor after interstitial pneumonia

**DOI:** 10.3389/fonc.2023.1126769

**Published:** 2023-03-14

**Authors:** Luo Wang, Haibo Mou, Xuehua Hou, Qin Liao

**Affiliations:** Department of Medical Oncology, Shulan (Hangzhou) Hospital Affiliated to Zhejiang Shuren University, Shulan International Medical College, Hangzhou, China

**Keywords:** case report, colon cancer, PD-L1 inhibitor, MSI-H, interstitial lung disease

## Abstract

Immune checkpoint inhibitors (ICI) have dramatically transformed the treatment landscape for metastatic colorectal cancer (mCRC) with deficient DNA mismatch repair (dMMR) or high microsatellite instability (MSI-H). Envafolimab, a novel programmed death-1 ligand 1 (PD-L1) inhibitor, has been reported to be efficient and safe for the management of advanced MSI-H/dMMR solid tumors. Here, we report the case of a 35-year-old female patient with MSI-H/dMMR mCRC who was treated with envafolimab following mFOLFOX6 (oxaliplatin, leucovorin, and fluorouracil) plus bevacizumab. While suffering from interstitial pneumonia after chemotherapy, the patient achieved a complete clinical response with the use of envafolimab without additional adverse events. Thus, PD-L1 inhibitors may be potential candidates for treating patients with MSI-H/dMMR mCRC.

## 1 Introduction

Colon cancer is the third most common cancer among males in the United States, with 151,030 new cases estimated for 2022. The median age at diagnosis has become younger (1). A genetic subset in 10%–20% of colorectal cancers has dMMR, which results in abundant neoantigens that initiate an immune response (2). According to the National Comprehensive Cancer Network guidelines, programmed death 1 (PD-1) inhibitors were recommended to treat patients with dMMR mCRC. However, there are no recommendations for those with dMMR who cannot afford financially imported drugs and are resistant to chemotherapy. A phase II randomized controlled trial proved a novel PD-L1 inhibitor, envafolimab, to be safe and effective in the treatment of patients with recurrent advanced MSI-H/dMMR solid tumors (3). Herein, we report a complete clinical response after envafolimab administration in a case of dMMR/MSI-H mCRC complicated by interstitial pneumonia during mFOLFOX6 plus bevacizumab treatment.

## 2 Case description

**Figure 1:**
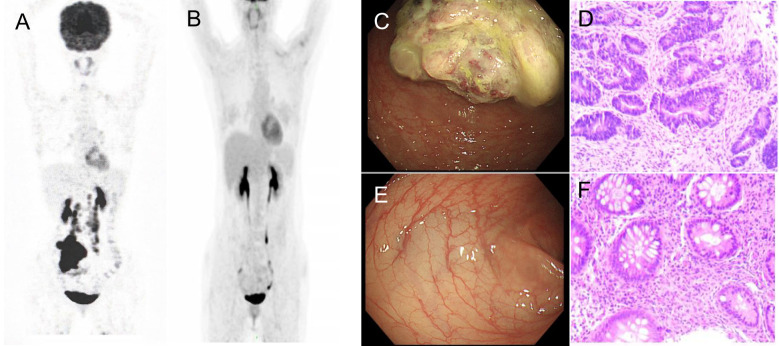
PET/CT and coloscopy findings at baseline and after treatment. PET/CT showed disease status **(A)** before treatment and **(B)** after treatment. **(C)** The coloscopy showed the tumor in the right colon. **(D)** Immunohistochemistry of pre-treatment biopsy confirmed tumor cells. **(E)** Post-treatment coloscopy did not display any neoplasm. **(F)** Pathologic analysis of the tissue manifested chronic mucositis.

A 35-year-old female complaining of abdominal discomfort and diarrhea for several months was referred to a local hospital. Abdominal computed tomography (CT) revealed a neoplasm located in the right colon, and positron emission tomography/computed tomography (PET/CT) showed a thickened nodule in the retroperitoneal area (**Figure 1A**). There was no significant family history of colorectal cancer or extraintestinal malignancies related to Lynch syndrome. This patient was admitted to our hospital for further treatment.

**Figure 2:**
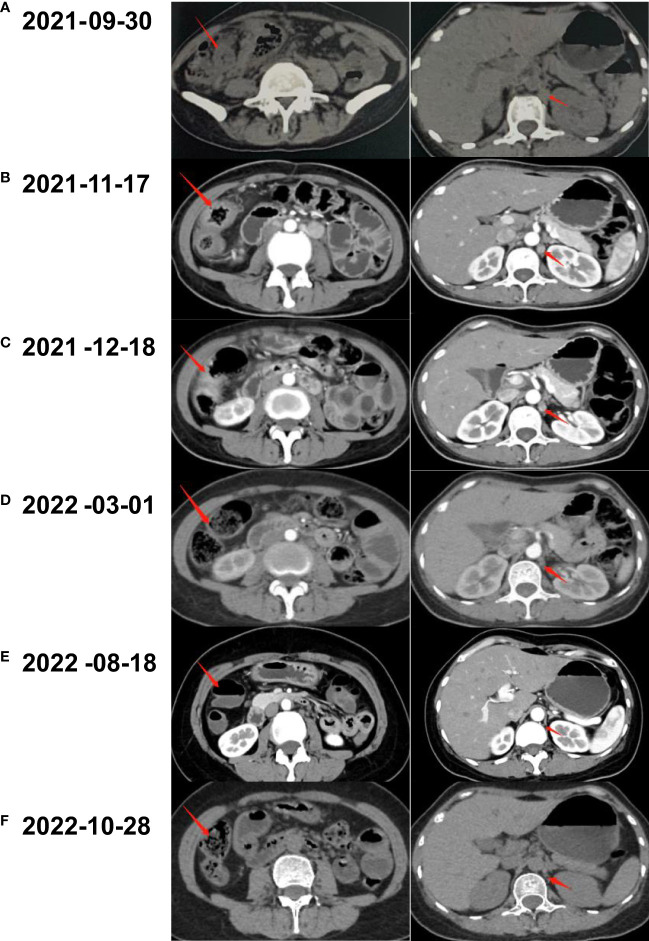
Abdominal CT scanning of the patient. **(A)** A neoplasm located in the right colon and thickened nodule in the retroperitoneal were shown by CT before treatment. **(B)** After three cycles of the combination of mFOLFOX6 and bevacizumab, CT demonstrated shrunken tumors in abdomen. **(C)** The lesion of colon and nodular did not continue to regress after another two cycles of chemotherapy plus targeted therapy. **(D)** CT scan revealed that the lesions came to gradual diminishment after eight cycles of envafolimab. **(E)** Thirty-six cycles of envafolimab later, the lesion in the right colon completely disappeared. **(F)** The patient kept clinical complete response with the use of envafolimab 400mg every 3 weeks.

**Figure 3:**
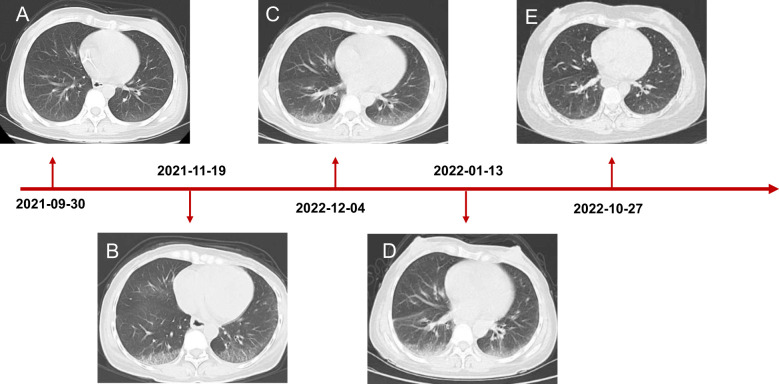
Chest CT scanning of the patient. **(A)** CT showed lung status without disease before treatment initiation. **(B)** The patient developed interstitial pneumonia after three cycles of mFOLFOX6 and bevacizumab. **(C)** CT demonstrated lung injury did not progress after five cycles of chemotherapy and targeted therapy. **(D)** Interstitial lung disease still existed but did not worsen after two cycles of envafolimab. **(E)** The lung disorder even reversed after the patient challenged envafolimab 400mg every three weeks.

**Figure 4:**
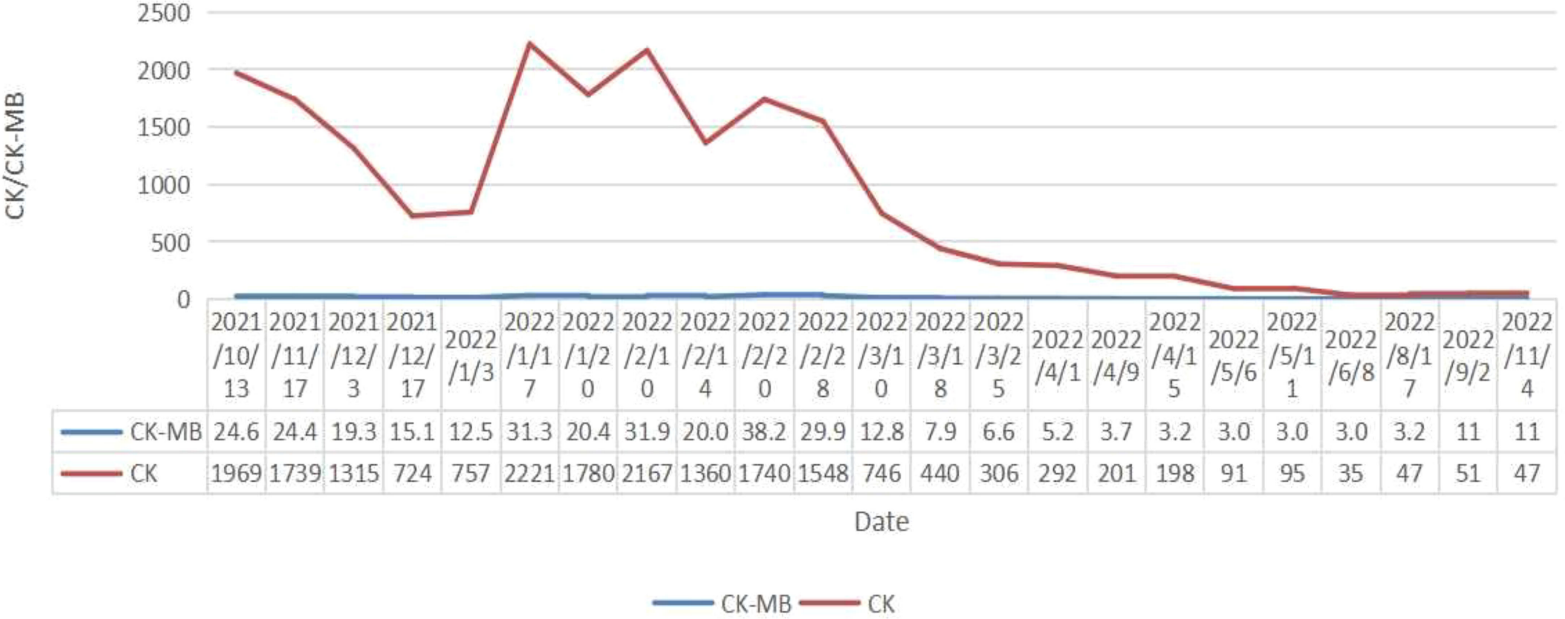
Changes of CK and CK-MB throughout the process of the treatment.

Coloscopy was performed, and pathological analysis of the tumor revealed adenocarcinoma (**Figures 1C, D**). Immunohistochemistry showed positivity for MLH1, MSH2, and MSH6 and negativity for PMS2. Next-generation sequencing confirmed that the tumor was MSH-H. However, the patient refused immunotherapy. Thus, a first cycle of chemotherapy consisting of mFOLFOX6 plus bevacizumab was administered on 1 October 2021. After three cycles, an abdominal CT scan showed an impressive reduction in tumor size in the right colon, while the retroperitoneal lesion remained stable. A chest CT revealed interstitial pneumonia (**Figure 2**), but the patient did not experience dyspnea. A multidisciplinary team (MDT) discussion, including surgical staff, was organized to formulate further treatment plans. Specialists recommended pembrolizumab, nivolumab, or nivolumab in combination with ipilimumab—all approved therapies for patients with metastatic MSI-H/dMMR colon cancer regardless of their eligibility for intensive therapy. Since the patient could not afford the expenses of the recommended courses of treatment, envafolimab was suggested instead. With patient and treatment teams reaching an agreement, the patient was started on envafolimab (150 mg, administered subcutaneously, once weekly) on 21 September 2021. Interstitial pneumonia did not worsen over the course of treatment, as evidenced by chest CTs. One month later, the patient experienced pain in her extremities with elevated creatine kinase (CK) and creatine kinase isoenzyme MB (CK-MB) levels. No significant laboratory findings involving cardiac troponins or N-terminal pro-brain natriuretic peptide were observed. We consulted cardiologists in our hospital. who excluded the possibility of ICI-related myocarditis but suspected myositis. However, she refused a proposal of muscle biopsy. According to the Management of Immune Checkpoint Inhibitor-Related Toxicity section of the Guidelines of the Chinese Society of Clinical Oncology, the symptom is classified in Grade 1, for which dose reduction or discontinuation are not considered. Therefore, nonsteroidal anti-inflammatory drugs (NSAIDs) and low-dose methylprednisolone (12 mg daily) were administered, and immunotherapy was continued. Decreased CK and CK-MB were observed three days after management (**Figure 3**). On 1 March 2022, a CT scan showed a decrease in volume, prompting a second MDT to suggest the patient continue treatment. A greater reduction in lesion size was observed during a follow-up CT scan in May 2022. Moreover, envafolimab with methylprednisolone (4 mg, daily) was well tolerated. Thirty-six cycles later, the lesion in the right colon disappeared on an abdominal CT scan performed on 18 August 2022 (**Figure 4**). Post-treatment colonoscopy revealed no neoplasm, and immunohistochemistry revealed chronic mucositis (**Figures 1E, F**). PET/CT also showed that the patient achieved CR efficacy (**Figure 1B**). Given the safety of envafolimab, the patient was started on 400 mg of envafolimab every 3 weeks without additional adverse events. CK and CK-MB levels remained low.

## 3 Discussion

The success of this case is underscored by recent evidence that MSI-H/dMMR solid tumors respond well to PD-L1 inhibitors, increasing survival. A recent clinical trial, KEYNOTE 177, showed that another PD-L1 inhibitor, pembrolizumab, resulted in remarkably longer median progression-free survival (PFS) in 307 patients with previously untreated dMMR mCRC (4). Moreover, the median overall survival found with pembrolizumab could not be replicated in the 367-month chemotherapy group. Thus, pembrolizumab successfully creates anti-tumor activity while having fewer adverse events than chemotherapy (5). Nivolumab, another PD-1 inhibitor, was studied in the CheckMate 142 clinical trial as monotherapy and presented a new option for patients with MSI-H/dMMR mCRC. Among the 74 patients who received treatment, 23 achieved an objective response, and 51 achieved tumor growth stagnation for more than 12 weeks (6). Nivolumab plus ipilimumab (a cytotoxic T-lymphocyte antigen-4 immune checkpoint inhibitor) as a first-line treatment or in previously treated patients also provided encouraging PFS and OS rates with manageable safety (7, 8). In addition to inhibition of the PD-1 pathway, the COMMIT study explored anti-PD-L1 atezolizumab monotherapy versus the mFOLFOX6/bevacizumab + atezolizumab combination. At the follow-up of 24 months, 48% of the patients in the control arm were free of progression (9). Besides that, envafolimab, the first subcutaneous PD-L1-targeting antibody, also gained encouraging results in the KN035-CN-006 study. There were 103 patients enrolled. 42.7% of them achieved an objective response, and the disease control rate reached 66.0% (3).

Due to the lack of data from randomized studies for PD-L1 inhibitors in MSI-H/dMMR mCRC, we may instead refer to other studies of PD-L1 blockade. For instance, the 5-year follow-up from the KEYNOTE-042 study showed that patients who completed 35 cycles of pembrolizumab were still alive four years after treatment. The continued use of pembrolizumab offers long-term benefits in OS and quality of life (10). The PACIFIC trial enrolled patients with stage III unresectable non-small cell lung cancer who had received concurrent chemoradiotherapy and were administered durvalumab for up to 12 months. This resulted in a sustained survival benefit of 42.9% in patients after 5 years. Thus, durvalumab was established as the standard of care at several sites (11). Moreover, in the Impower010 study, patients were randomly assigned to receive atezolizumab for 16 cycles, or 1 year, or best supportive care. These findings suggested that those in the atezolizumab treatment group achieved significantly improved disease-free survival (DFS) compared to standard care after a median follow-up of 322 months (12). Altogether, the use of PD-L1 inhibitors in MSI-H/dMMR mCRC may extend survival by at least 1 year; however, further investigation is warranted.

The success of nivolumab in CheckMate 142 implies that chemotherapy may increase the immunogenicity of tumor cells in patients receiving anticancer agents including oxaliplatin and 5-fluorouracil (5FU) before immunotherapy, inducing cells to secrete, release, and expose surfaces when dying, stressed, or injured. The molecules produced during this process are called damage-associated molecular patterns (DAMPs), which are crucial for the immunogenic cell death (ICD) of tumor cells. Interestingly, oxaliplatin can augment immunogenicity by directly and indirectly triggering ICD by releasing DAMPs (13). Chemotherapy, including 5FU, may also have an antitumor effect through its selective action on myeloid-derived suppressor cells (MDSC). Both *in vitro* and *in vivo* studies have indicated that 5FU is strongly cytotoxic to MDSC, which mediates immune tolerance. The decrease in MDSC stimulates CD8(+) T cells to produce IFN-γ; thus, CD8(+) T cells could also play a role in promoting anti-tumor responses (14).

In this case, our patient developed interstitial pneumonia after two cycles of mFOLFOX6 and bevacizumab. While the component causing pulmonary toxicity was unknown, a few reports have found bevacizumab and leucovorin to cause lung damage (15, 16). Conversely, oxaliplatin may have been the toxic agent, as the pulmonary disease did not recur with oxaliplatin-free chemotherapy but did with 5FU and leucovorin (17, 18). Though pulmonary toxicity from FOLFOX is uncommon, it could be lethal despite the discontinuation of the agents and the initiation of immunotherapy. Considering the reasonable safety of envafolimab, with no immune-related pneumonitis in its phase 2 study, it was used to treat the patient (3). Fortunately, the interstitial pneumonia did not worsen. Our patient was administered a subcutaneous injection of 300 mg Q3W when CK and CK-MB trended toward stability. Moreover, the fixed-dose schedule prevented additional adverse events and proved efficacious and convenient. Notably, the patient also developed immunotherapy-induced myositis, a rare condition typically only seen in a case series. While immune-related myositis was not observed in the envafolimab clinical trial, it has been reported as a complication of other ICIs. Among 9,088 patients receiving ICIs at the MD Anderson Cancer Center, 0.4% were diagnosed with ICI-myositis, 0.94% with combination ICI therapy, and 0.31% with ICI monotherapy. The delay between ICI initiation and myositis onset was approximately one month. Moreover, the onset of myalgia during ICI-myositis was quicker than that in primary autoimmune polymyositis (19). Reported symptoms characteristic of ICI-myositis include myalgia, limb-girdle weakness, and oculomotor weakness with diplopia (20). The outcomes of patients with only elevated CK, including those who needed short hospitalizations for CK normalization, were more favorable than those of patients with overlapping syndromes (21). Further studies are needed to clarify the mechanism of ICI-myositis, the biomarkers to predict, and the time to rechallenge ICIs.

With respect to the biomarkers of progressive disease for MSI-H/dMMR tumors, the related data is still inconclusive so far. Some studies suggest that a higher number of mutation-associated neoantigens due to mismatch-repair deficiency is associated with improved anti-PD-1 responsiveness in dMMR cancers, regardless of the origin of the cancerous tissue of the cancers (22, 23). *B2M* mutations that could impair antigen presentation were widely seen in MSI-H CRCs. It was found that the mutations of *B2M* and the loss of protein expression may also make patients obtain benefit from immunotherapy (24). It is noteworthy that discrepant results between immunohistochemistry and polymerase chain reaction may occur when determining the status of MSI or dMMR (25). Misdiagnosis of the MSI or dMMR status can be the direct cause of resistance to immune checkpoint inhibitors. Therefore, the two methods mentioned above should be routinely performed.

In conclusion, envafolimab is an effective and safe PD-L1 inhibitor for dMMR/MSI-H mCRC. The importance of monitoring and identifying ICI-myositis and interstitial pneumonia, which are important indications for chemotherapy treatment, should be highlighted. Further research is warranted to guide physicians in determining optimal treatment.

## Data availability statement

The original contributions presented in the study are included in the article/supplementary materials. Further inquiries can be directed to the corresponding author.

## Ethics statement

Written informed consent was obtained from the individual(s) for the publication of any potentially identifiable images or data included in this article.

## Author contributions

LW collected data and draft the manuscript. QL and the rest of the authors revised the manuscript and approved the final version. All authors contributed to the article and approved the submitted version.
